# High-to-Low CO_2_ Acclimation Reveals Plasticity of the Photorespiratory Pathway and Indicates Regulatory Links to Cellular Metabolism of Arabidopsis

**DOI:** 10.1371/journal.pone.0042809

**Published:** 2012-08-17

**Authors:** Stefan Timm, Michael Mielewczik, Alexandra Florian, Silja Frankenbach, Anne Dreissen, Nadine Hocken, Alisdair R. Fernie, Achim Walter, Hermann Bauwe

**Affiliations:** 1 University of Rostock, Department of Plant Physiology, Rostock, Germany; 2 Institute of Agricultural Sciences, ETH Zürich, Zürich, Switzerland; 3 Institute of Bio- and Geosciences IBG-2, Forschungszentrum Jülich GmbH, Jülich, Germany; 4 Max Planck Institute of Molecular Plant Physiology, Potsdam-Golm, Germany; Purdue University, United States of America

## Abstract

**Background:**

Photorespiratory carbon metabolism was long considered as an essentially closed and nonregulated pathway with little interaction to other metabolic routes except nitrogen metabolism and respiration. Most mutants of this pathway cannot survive in ambient air and require CO_2_-enriched air for normal growth. Several studies indicate that this CO_2_ requirement is very different for individual mutants, suggesting a higher plasticity and more interaction of photorespiratory metabolism as generally thought. To understand this better, we examined a variety of high- and low-level parameters at 1% CO_2_ and their alteration during acclimation of wild-type plants and selected photorespiratory mutants to ambient air.

**Methodology and Principal Findings:**

The wild type and four photorespiratory mutants of *Arabidopsis thaliana* (Arabidopsis) were grown to a defined stadium at 1% CO_2_ and then transferred to normal air (0.038% CO_2_). All other conditions remained unchanged. This approach allowed unbiased side-by-side monitoring of acclimation processes on several levels. For all lines, diel (24 h) leaf growth, photosynthetic gas exchange, and PSII fluorescence were monitored. Metabolite profiling was performed for the wild type and two mutants. During acclimation, considerable variation between the individual genotypes was detected in many of the examined parameters, which correlated with the position of the impaired reaction in the photorespiratory pathway.

**Conclusions:**

Photorespiratory carbon metabolism does not operate as a fully closed pathway. Acclimation from high to low CO_2_ was typically steady and consistent for a number of features over several days, but we also found unexpected short-term events, such as an intermittent very massive rise of glycine levels after transition of one particular mutant to ambient air. We conclude that photorespiration is possibly exposed to redox regulation beyond known substrate-level effects. Additionally, our data support the view that 2-phosphoglycolate could be a key regulator of photosynthetic-photorespiratory metabolism as a whole.

## Introduction

Oxygenic photosynthesis requires operation of the photorespiratory pathway to recycle 2-phosphoglycolate (2PG), the major by-product of Rubisco, to 3-phosphoglycerate (3PGA; recently reviewed in [1], [2]). One of the enzymatic reactions of this pathway releases NH_3_ and CO_2_, which re-enter metabolism more (NH_3_) or less (CO_2_) completely. Whilst 2PG production can be reduced via CO_2_-concentrating mechanisms such as those occurring in green algae and C_4_ plants, photorespiration cannot be fully avoided and occurs with high rates in most land plants. As a result, net-CO_2_ assimilation is reduced in comparison with artificial low-O_2_ environments. The rates of photorespiration do not only depend on the concentrations of O_2_ and CO_2_ but also on other environmental parameters. Well-studied effects are, for example, the promotion of photorespiration by high temperatures [3]–[5] and high light intensities [6], which is thought to help in the thermal and high-light protection of plants [7]. Photorespiration is also the major source of H_2_O_2_ in plants, a compound that is involved in stress response signalling [2], [8] and pathogen defence [9]. In light of this complexity and since environmental factors interact and fluctuate on small timescales, such as hours to days, highly differentiated responses of photorespiratory metabolism to such changes are likely.

Photorespiration is thus a multifaceted process. It allows oxygenic photosynthesis by recycling 2PG, which is the indispensable function of this pathway, but it also interacts with several other cellular processes and responds to changes in the environment. The important role of photorespiration is most directly apparent from mutant studies in diverse organisms, ranging from cyanobacteria [10] to a variety of land plants [11]–[13]. Among these, *Arabidopsis thaliana* (Arabidopsis) is the only plant for which a comprehensive set of genetically well characterized photorespiratory knockout mutants exists. Several of these mutants were produced in the 1970s by chemical mutagenesis [14] and many more became available from the advent of T-DNA insertional mutagenesis [15]. With the possible exception of glycolate oxidase, where the existence of five isoforms hampered studies, this set covers all known reactions of the photorespiratory core cycle and also includes a range of associated reactions.

Traditionally, photorespiratory mutants are referred to as displaying a ‘photorespiratory phenotype’. That is, they do not survive in normal air but can be recovered in air enriched to 1% CO_2_ [16]. The examination of available growth data shows, however, that only some but not all photorespiratory mutants require such high CO_2_ levels. A high level of CO_2_, for example, is required for normal growth of mutants devoid of 2PG phosphatase (PGLP) [17], [18], [19], whereas null mutants of glycerate 3-kinase (GLYK) grow well with about 0.15% CO_2_ [20]. Mutants without peroxisomal hydroxypyruvate reductase (HPR1) can even grow and reproduce in normal air [21], [22]. Since all three mutants are defective in single-gene-encoded enzymes of the core photorespiratory cycle in Arabidopsis, their differential CO_2_ response indicates a much higher complexity of photorespiratory metabolism than presently assumed.

In this article, we examine this complexity by investigating the acclimation of plants grown in low-photorespiratory (1% CO_2_, HC for high CO_2_) conditions to the high-photorespiratory conditions of (0.038% CO_2_, LC for low CO_2_). We found considerable variability in the acclimation of the individual photorespiratory mutants and in the wild type. Most of these differences depend on the position of the impaired enzymatic step in the pathway and are imprinted in the diel (24 h) growth dynamics. Acclimation was typically steady and consistent for a number of features, but we also found some unexpected transition events, such as an intermittent very massive rise of glycine levels after transition of the HPR1-deficient mutant to ambient air. We will discuss these data with focus on possible regulatory interactions between photorespiration and other major metabolic pathways.

## Results

For our study, we selected four T-DNA insertional mutants of Arabidopsis: (1) *pglp1*, a null mutant of the chloroplastidial enzyme phosphoglycolate phosphatase (PGLP) [17], which is the first enzyme of the photorespiratory pathway; (2) *shm1*, a null mutant of the mitochondrial enzyme serine hydroxymethyltransferase 1 (SHM1) [23]; (3) *hpr1*, a null mutant of the peroxisomal enzyme hydroxypyruvate reductase 1 (HPR1) [21], [24]; and (4) *glyk1*, a null mutant of the chloroplastidial enzyme glycerate 3-kinase (GLYK) [20], which is the final enzyme of the photorespiratory pathway. To ensure the best comparability possible, all plants were grown to developmental stage 5.1 [25] in conditions that are normally classified as ‘non-photorespiratory’ (HC, 1% CO_2_; initial growth conditions). Then, keeping all other growth parameters unchanged, we reduced the CO_2_ level to ambient concentration and monitored transitional effects that occurred during acclimation to ambient-air conditions (LC, 0.038% CO_2_) for a variety of parameters. Since distinct day-length effects exist with respect to the growth of some photorespiratory mutants, for example catalase (CAT2) [26] and HPR1 [21], [24], all experiments were performed in strictly controlled short-day conditions. This approach triggered the very weak effects typically observed in long-day-grown *hpr1* and moderated the very strong effects in the other three mutants typically observed in long days (as illustrated in Figure S1and Figure S2) to allow analysis of growth and photosynthesis in leaves that are not damaged to an extent that precludes an exact analysis.

### Photorespiratory Mutants Display Different Phenotypes

**Figure 1:**
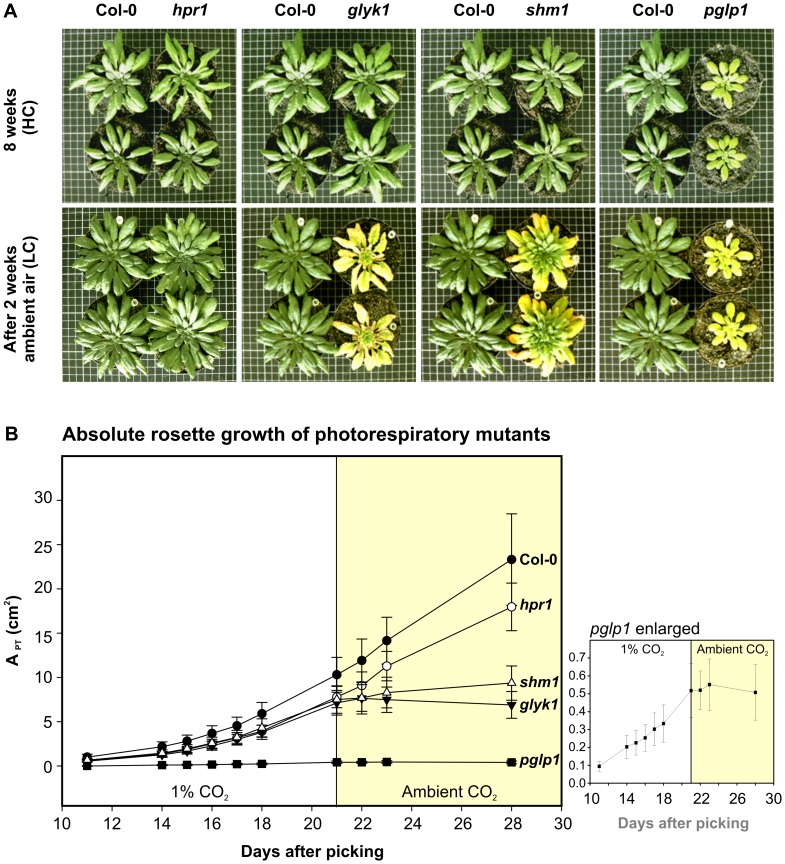
Phenotypes and growth performances at high CO_2_ and after transition to ambient air. (**A**) Plants were first grown in 1% CO_2_ (HC) for eight weeks to stadium 5.1 [25], then transferred to ambient air (LC; 0.038% CO_2_) and monitored for another 2 weeks. For more detailed time courses see Figure S1 and Figure S2. (**B**) Absolute rosette growth rates of photorespiratory mutants before and after transfer to LC. Shown is the projected rosette area (A_PT_) as calculated from automated image acquisition and analysis using the phenotyping platform GROWSCREEN FLUORO. Plants were transferred to LC before reaching stadium 5.1 to allow comparability with the DISP-based growth analysis (shown in Figure 5) of young developing leaves before and after transfer. Monitoring started 11 days after picking for a period of 10 days in HC conditions (stadium 1.04). Next, plants were transferred to LC and monitored for another 7 days. Data points represent mean values ± SD from 8 individual plants.

We first wanted to obtain an overview over the phenotypic differences between the individual mutants and in comparison with the wild type (Figure 1A). At HC, there were no visible differences between the wild type, *hpr1*, *glyk1*, and *shm1*, whereas the *pglp1* mutant already displayed yellowish leaves and growth retardation. After several days at LC, clear alterations were visible in the cases of *glyk1* and *shm1* and even stronger in the case of *pglp1*. By PSII fluorescence imaging (as shown in Figure S3), alterations were detectable in all four mutants already after three days at LC. As earlier reported for corresponding barley [22] and Arabidopsis mutants [21], the *hpr1* mutant reacted much more mildly compared to the other three mutants. This very moderate response has been explained by extraperoxisomal bypasses [21], [22]. Interestingly, source leaves of *glyk1* and *shm1* were more strongly impaired by the induction of photorespiration than sink leaves. This heterogeneous phenotype was not observed before and most distinctive in the *shm1* mutant, whose younger leaves stayed green and photosynthetically active while the older leaves became increasingly necrotic. Interestingly, though to a much lesser extent, even the highly impaired PGLP null-mutant displayed some long-term acclimation: following initial growth at HC, this mutant survived for up to three weeks in ambient air.

In addition to this visual inspection, absolute growth performances were calculated by automated image acquisition and analysis using the phenotyping platform GROWSCREEN FLUORO [27] (Figure 1B). Underpinning the visible effects, rosettes of *pglp1* showed a reduced absolute growth rate already under HC conditions, whereas *hpr1*, *shm1* and *glyk1* displayed comparable growth rates at HC if compared to the wild type. By contrast, after plants were exposed to ambient air, clear differences in absolute growth rates became visible in the order wild type >*hpr1*>*shm1*∼*glyk*>*pglp1*.

### Different Mutations Have Different Impact on PSII Photochemical Efficiency

**Figure 2:**
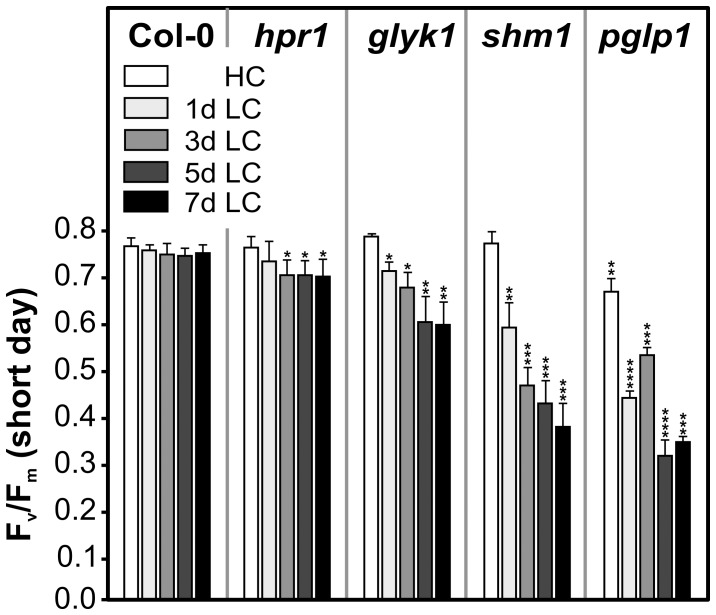
Changes in PSII photochemical efficiency F_v_/F_m_ in response to ambient air. Plants were grown at HC and transferred to ambient air as described in the legend to Figure 1A. F_v_/F_m_ ratios were measured before (HC control) and after transfer to normal air (LC; 1, 3, 5 and 7 days). At the indicated times after transfer, plants were dark-adapted for 10 min and F_v_/F_m_ values determined from fully expanded individual source leaves. Mean values ± SD (n = 5) are shown. Asterisks show significant alterations according to the two-tailed Student's *t*-test (p<0.05, * to wild-type, ** to *hpr1*, *** to *glyk1*, **** to *shm1*).

Photorespiration consumes ATP and reducing equivalents, both directly within the photorespiratory carbon and nitrogen cycles and indirectly via effects on the Calvin-Benson cycle. Consequently, due to the high fluxes, perturbation of photorespiration accelerates photoinhibition [28], and it was also reported that repair of the PSII D1 protein becomes less efficient [29]. We assessed PSII maximum efficiency (F_v_/F_m_) of fully expanded individual leaves of dark-adapted plants (from the same set as shown in Figure 1) prior to and following HC-to-LC transition (Figure 2). In close correspondence to the visual appearance of the HC-grown plants, the F_v_/F_m_ ratios of individual leaves were similar in wild type, *hpr1*, *glyk1* and *shm1* but significantly reduced in *pglp1*. However, slight but distinct impairments were also visible at the edges of younger rosette leaves of *hpr1*, *glyk1* and *shm1* (Figure S3). Transition to ambient CO_2_ had little effect on F_v_/F_m_ of the *hpr1* mutant, whereas F_v_/F_m_ became rapidly, strongly and increasingly reduced in the other three mutants. Generally described, the effects of the knockouts on F_v_/F_m_ followed the order *hpr1*<*glyk1*<*shm1*<*pglp1*.

**Figure 3:**
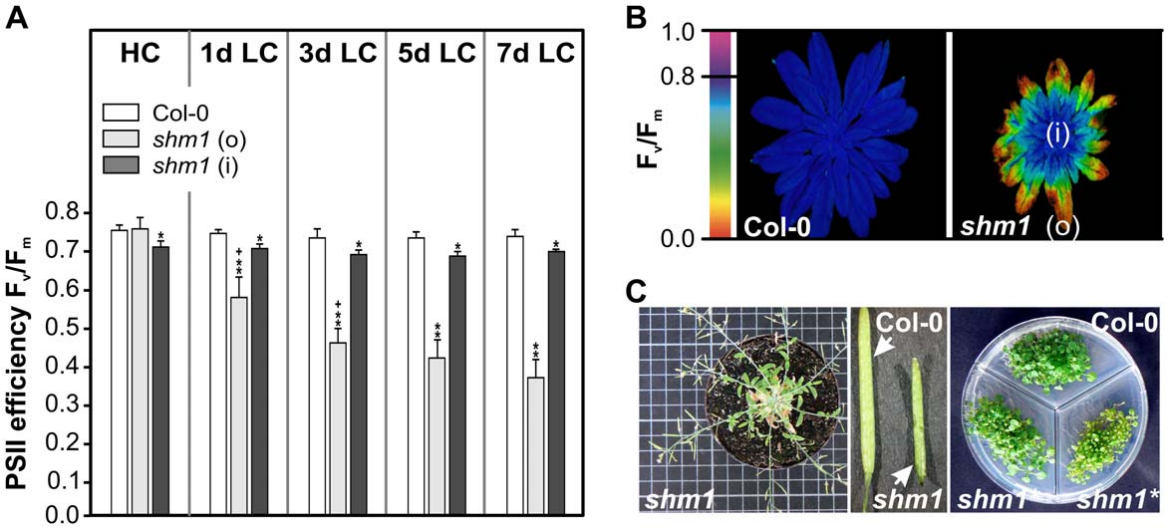
The *shm1* mutant shows long-term acclimation and survives if initially grown in high CO_2_. (**A**) PSII efficiency (F_v_/F_m_) rapidly decreases after transfer to ambient air in source leaves but not in sink leaves. Mean values ± SD (n = 5) are shown. Asterisks mark the significance of alterations according to the two-tailed Student's *t*-*test* (p<0.05, * to wild type, ** to *shm1* outer area of source leaves, and ^+^ to the day before). (**B**) PSII fluorescence imaging of a *shm1* rosette illustrating acclimation of sink leaves with a clear gradient toward better performance of the youngest leaves (i) but not of source leaves (o) after two weeks in ambient air. A wild-type rosette is shown for comparison. (**C**) After transfer to LC, *shm1* produces new sink-leaves, flowers, siliques, and fertile seeds. The right photo shows a germination test on ½ MS media, compared to wild-type and *shm1* seeds collected from plants grown produced under elevated CO_2_. shm1* labels germinated seeds from *shm1* plants grown under HC conditions throughout their entire life cycle.

In light of the clear differences between sink and source leaves of *shm1*, we extended the examination of this particular mutant beyond two weeks after transition to ambient air. Within the first week after HC-to-LC transition, F_v_/F_m_ decreased strongly in the source leaves but remained nearly unaltered in the sink leaves and close to wild-type levels (Figure 3A and B). Thereafter, the *shm1* mutant unexpectedly continued to produce new sink leaves and eventually even flowered and produced fertile seeds (Figure 3C).

### Photorespiratory Mutants Display Differing Alterations in Leaf Gas Exchange

**Figure 4:**
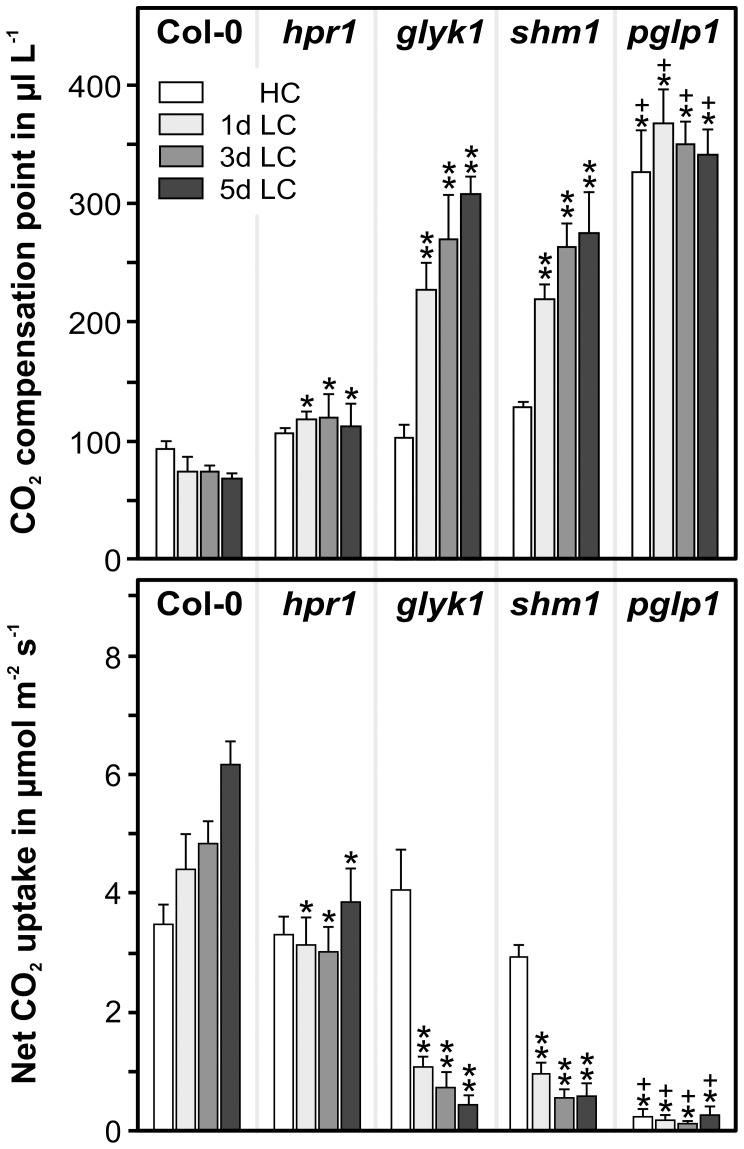
Alteration of gas exchange after transfer from high CO_2_ to ambient air. Plants were grown in HC and transferred to LC exactly as described in the legend to Figure 1 (short day conditions). Net CO_2_ uptake (A, at 380 µl L^−1^ CO_2_) and CO_2_ compensation points (Γ_21_) were measured before (HC control) and after (LC; 1, 3 and 5 days) transfer to normal air. Mean values ± SD (n = 5) are shown. Asterisks show significant alterations according to the two-tailed Student's *t*-test (p<0.05, * to wild-type, ** *to hpr1*, ^+^ to *glyk1* and *shm1* at the corresponding time points).

Perturbations of photorespiratory metabolism alter leaf gas exchange, which is a useful proxy for photosynthetic performance. We therefore examined the acclimation of net CO_2_ uptake rates and CO_2_ compensation points of fully expanded leaves, both prior to the HC-to-LC transition and at days 1, 3 and 5 thereafter (Figure 4). After transition to ambient air, the wild type slowly but progressively acclimated as visible in lowered CO_2_ compensation points (Γ_21_) and increasingly improved net-CO_2_ uptake rates (A). Such acclimation was not observed with *hpr1*, and minor changes in both parameters indicated that photorespiratory metabolism is already slightly disturbed in this mutant. Much clearer incremental changes were observed with leaves of *glyk1* and *shm1*. These very similarly responding mutants displayed marked negative effects on gas exchange already one day after transfer to ambient CO_2_, followed by more gradual alterations during the subsequent four days. The *pglp1* mutant behaved differently: while both the CO_2_ uptake and compensation point differed very strongly from the respective wild-type values already at HC, they deteriorated much less in the subsequent low-CO_2_ environment and even re-improved slightly though significantly between days 1 and 5.

Since photosynthesis interacts closely with carbohydrate metabolism, it is not surprising that changes in the biosynthesis and contents of major carbohydrates have been observed in photorespiratory mutants, for example [30]. Moreover, sugars contribute to the regulation of many diurnally expressed controlled genes and control diel changes of leaf growth [31]. Preliminary studies (not shown) indicated that neither of the examined mutants showed any clear alterations in leaf starch content or the typical diurnal rhythm of starch synthesis and starch degradation at HC [31]–[33]. We hence quantified leaf starch contents two days after HC-to-LC transition to allow some metabolic re-adjustment. Under this condition in ambient air, *hpr1* and *pglp1* accumulated less starch than the wild type within the first 5 h of illumination (Table 1, mid of day). This trend continued over the day to include all mutant lines over the next 5 h in light (Table 1, end of day). The effect on starch accumulation was most pronounced in *pglp1*, in which starch accumulated only to an extremely low level. This feature was accompanied by more than five-fold reduced nocturnal levels of maltose (Table S3), the major disaccharide generated during starch degradation and subsequently exported to the cytosol [34].

**Table 1:** Leaf starch contents after transition from high CO_2_ to ambient air.

Starch contents in µmol Glc g^−1^ fresh weight ± SD			
	EON	MOD	EOD
Col-0	1.56±0.27	18.6±3.96	65.9±19.1
*hpr1*	1.68±0.46	**11.6±4.57**	**28.6±12.5**
*shm1*	**5.31±1.84**	**24.2±1.72**	39.0±14.1
*glyk1*	2.25±2.83	18.1±7.86	**35.6±15.7**
*pglp1*	2.23±1.50	**3.71±3.15**	**8.1±9.1**

Plants were grown in a 10/14 h day/night cycle in HC. After reaching growth stadium 5.1 [25], CO_2_ was reduced to LC (ambient air levels). After two days in LC, leaf samples were harvested at the end of the night (EON), the middle (MOD) and the end of the day (EOD). Shown are mean values ± SD (n = 5). Values in bold are significantly different from the LC wild-type control according to the two-tailed Student's *t*-test (p<0.05).

### Photorespiratory Mutants Show Differentially Altered Diel Growth Patterns

**Figure 5:**
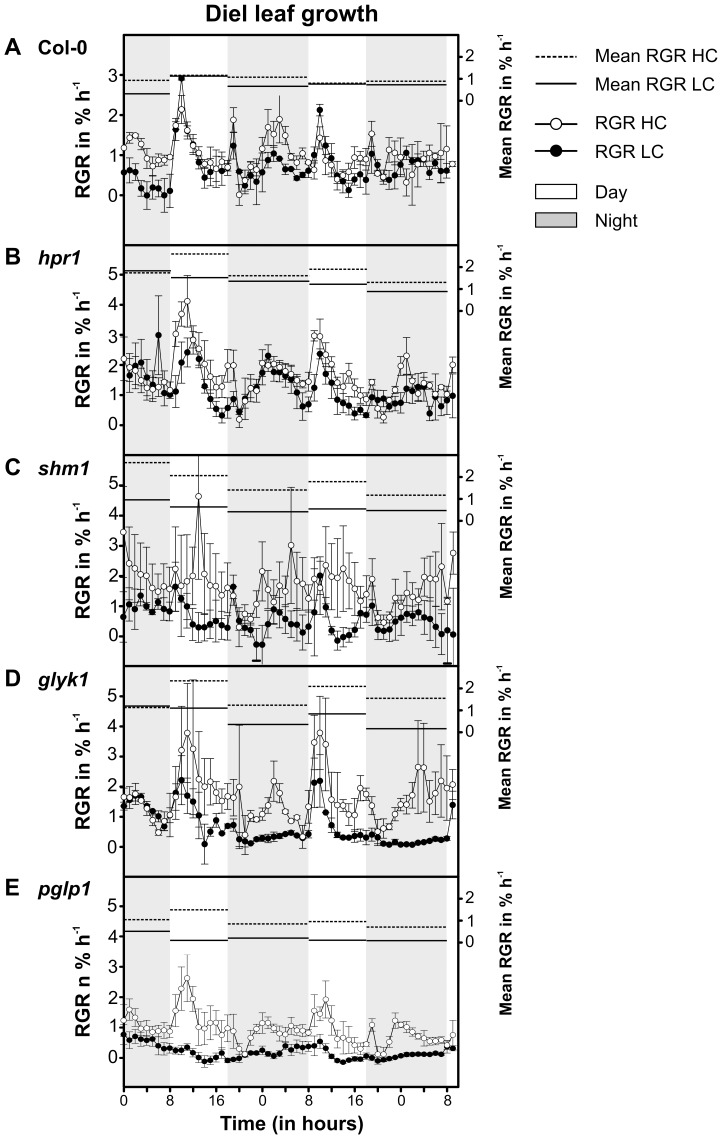
Diel (24 h) leaf growth patterns in high CO_2_ and thereafter in ambient air. Grey (night) and white (day) areas mark the 10/14 h photoperiodic phases. Relative growth rates (RGR, left scale) of the Arabidopsis wild type (A) and photorespiratory mutants *hpr1* (B), *shm1* (C), *glyk1* (D), and *pglp1* (E) in HC conditions (1% CO_2_, open circles) and after HC-to-LC transition in ambient CO_2_ (closed circles). Each data point represents the mean RGR ± SE of one-hour average RGR values of the preceding hour individually measured with five leaves, each from another plant. Mean RGR values (RGR averaged over the preceding hour) over the day and night period are shown as horizontal lines (right scale; HC, dashed lines; LC, solid lines).

While growth data similar to those shown in Figure 1B have occasionally been reported for individual mutants in the photorespiratory pathway, the quantitative diel (24 h) growth patterns and the dynamics of leaf-growth rates of such mutants are not known. We performed such analyses by digital image sequence-processing (DISP) [31] for both high- and low-CO_2_ conditions at a high temporal resolution. Figure 5A shows diel growth patterns (relative growth rates, RGR) of individual leaves of wild-type plants before and after HC-to-LC transition. In HC (white circles), leaf growth was highest during the early morning hours and lowest in the first hours of the night. After transfer to ambient CO_2_ (black circles), the observed pattern remained essentially unchanged. The mean wild-type RGR (RGR averaged over the day or night) of about 1% h^−1^ remained unaffected in comparison of day and night rates but was distinctly lower in LC than in HC during the night. These control data are almost identical to some earlier results for Arabidopsis [31], [35] and hence validate the experimental setup. The *hpr1* mutant (Figure 5B) showed a very similar diel growth pattern, with the exception of a significantly about 50% reduced RGR in the afternoon at LC. Much more pronounced alterations in the LC diel-growth patterns were observed in *shm1* and *glyk1* (Figure 5C and D). Similar to *hpr1*, *shm1* and *glyk1* displayed a considerably depressed growth in the afternoon but, in addition, nocturnal leaf growth was also distinctly lower than in *hpr1*. This effect was most pronounced in *glyk1*, where the differences between the average growth rates at day vs. night were distinctly higher than in the wild type. PGLP-knockout plants also showed a wild-type-like diel growth pattern in HC (Figure 5E) although overall leaf growth was slower than in the wild type and the other three mutants. Immediately after transition to LC, both the early-morning peak RGR and the mean RGR declined very strongly.

### Metabolite Responses in the Wild Type

The gradual alteration of leaf gas exchange parameters demonstrates that acclimation to low CO_2_ is not a fast process but requires several days. To start understanding what exactly happens after high-to-low CO_2_ transition at the metabolite level, we examined the metabolic re-adjustment of the wild type and the two ‘extreme’ mutants, *hpr1* and *pglp1*, using a metabolite profiling approach. The sampling intervals were the same as for the PSII and gas exchange measurements (developmental stage 5.1 at HC, after 1, 3 and 5 days at LC) with daily sampling times 2 h before and 4 h after switching off illumination. Relative steady-state levels of selected metabolites were analysed using gas-chromatography/mass-spectrometry (GC/MS) using wild-type values at HC as reference (individually set to 1 for both day and night values).

**Figure 6:**
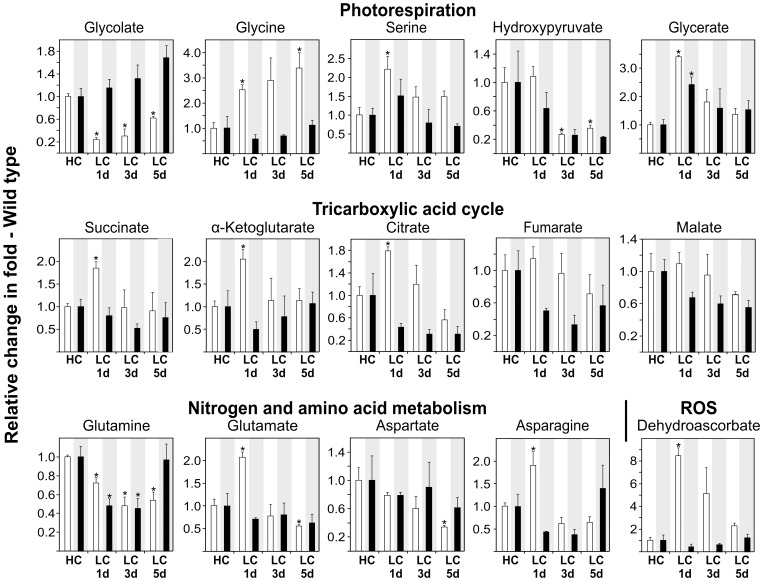
Metabolite responses of the wild type after transition to ambient air. Plants were grown in HC and transferred to LC with a continuous short-day photoperiod of 10/14 h throughout the experiment. Leaves of three individual plants were harvested 2 h before and 4 h after light was switched off in high-CO_2_ conditions (HC) and 1, 3 and 5 days after HC-to-LC transition. Relative metabolite contents ± SD are shown with the corresponding wild-type content at HC arbitrarily set to 1, separately for day and night values (white bars, day values; black bars, night values). Asterisks show significant changes to the corresponding wild-type HC control according to the two-tailed Student's *t*-test (p<0.05). The full data set is shown in Table S1.

In the wild type, on the background of a wide range of unchanged metabolites after HC-to-LC transition shown in Table S1, some distinct short- and long-term responses became obvious (Figure 6). Most of the changes expectedly concerned photorespiratory intermediates and metabolites of pathways already known or presumed to closely interact with photorespiration, namely nitrogen metabolism [36] and the tricarboxylic acid (TCA) cycle [37]. Not all metabolites with altered contents in the leaves though displayed the same accumulation pattern. For example, day glycolate levels displayed an unexpected sharp drop on day one after transition from low- to high-photorespiratory conditions and only slowly increased thereafter. It was also unexpected that glycolate levels were only decreased in light but not in the dark, where they became increasingly higher relative to HC over five days in ambient air. Similar to glycolate, but without the initial drop during the day and the steady increase during the night, the steady-state content of glycine in the light was distinctly increased one day after transfer to low CO_2_ and continued to increase during the following days. The corresponding day contents of serine and glycerate were also considerably elevated on day one in LC, but these massive initial rises of serine and glycerate successively deteriorated again. Interestingly, hydroxypyruvate levels changed in a different manner: day levels did not immediately respond to the LC environment but gradually decreased to about 30% of the HC reference level, that is, at *low*-photorespiratory conditions.

Not only the photorespiratory metabolites but also the TCA cycle intermediates respond to the HC-to-LC transition. In the light, contents of succinate, α-ketoglutarate and citrate become distinctly elevated one day after transition, whereas fumarate and malate day levels remained unaffected. During the following days, these metabolites adjusted to LC steady-state levels, which were unchanged (succinate, α-ketoglutarate) or lower (citrate, fumarate, malate) relative to HC conditions. Nitrogen and amino acid metabolism, as evidenced by changes in glutamate and asparagine, was also affected by the massive drop of CO_2_ concentration. Whilst most of the changes described above are not very great in a quantitative sense, very large initial increases with a subsequent slow decrease were observed for dehydroascorbate. These changes only occurred in illuminated leaves and were not observed in the dark period.

### Transient Accumulation of Glycine in *hpr1* Indicates Regulation of Glycine-into-Serine Conversion

**Figure 7:**
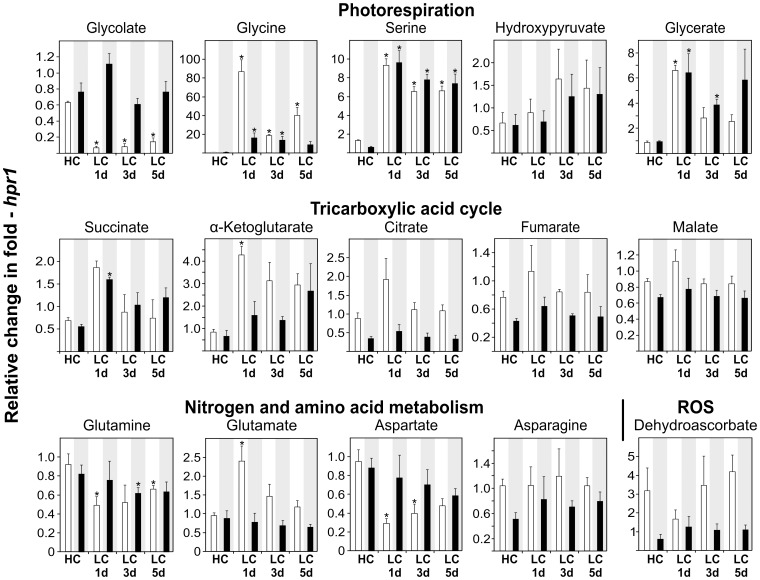
Metabolite responses of *hpr1* after transition to ambient air. Relative metabolite contents ± SD are shown with the corresponding wild-type content at HC arbitrarily set to 1 (white bars, day values; black bars, night values). See the legend to Figure 6 for more experimental details and Table S2 for the full data set.

As already mentioned, *hpr1* is an example for a relatively mildly perturbed photorespiratory cycle. The metabolite dynamics shown in Figure 7 (detailed list in Table S2) demonstrates that the knockout of HPR1, despite its only small effect on growth in ambient air, requires considerable more readjustment of metabolism after HC-to-LC transition than the wild type. Glycolate levels were somewhat lower in high CO_2_ than in the wild type at HC, but the initial drop under illumination in low CO_2_ was even more distinct than in the wild type, followed by a continuous slight rise. In the dark, glycolate levels were somewhat lower than in the wild type but still much higher than in the light. Glycine displayed a massive initial rise on day one after transition to low CO_2_ in the light (about eighty-fold) and also in the dark (about twenty-fold). This was followed by a sharp decrease on day three and another rise on day five in LC. Night levels of glycine decreased steadily but were still more than ten-fold higher than in the wild type after five days. Serine and glycerate levels also considerably increased in ambient air. This effect was strongest on day one but persistent throughout the experiment and also through the nights. In comparison with these strong effects, the initial transitional effect on hydroxypyruvate was surprisingly small with a mild increase over time in low CO_2_, which is opposite to the decrease observed in the wild type and can be explained by the absence of HPR1.

In the TCA cycle, distinct differences relative to the wild type were observed only with respect to higher levels of α-ketoglutarate in low CO_2_, both during the day and night, whereas other intermediates remained unchanged. Similarly, this mutant did not behave significantly different to the wild type with respect to key metabolites of nitrogen metabolism. That said, the three-fold elevated levels of dehydroascorbate at HC compared with a lower daily increase at LC could indicate that HPR1 contributes to peroxisomal redox homeostasis beyond photorespiration. A recently reported example is NADH-reoxidation during fatty acid β-oxidation [38].

### Knockout of PGLP1 Displays Links to TCA cycle and Metabolism of Branched-Chain Amino Acids

**Figure 8:**
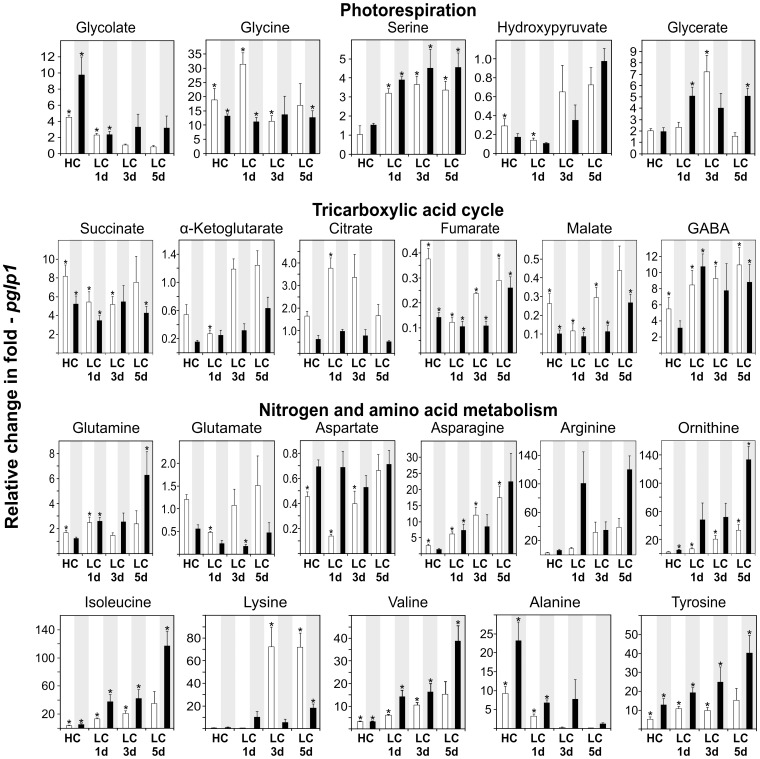
Metabolite responses of *pglp1* after transition to ambient air. Relative metabolite contents ± SD are shown with the corresponding wild-type content at HC arbitrarily set to 1 (white bars, day values; black bars, night values). See the legend of Figure 6 for more experimental details and Table S3 for the full data set.

*pglp1* represents the other extreme of photorespiratory phenotypes and responds very strongly to low-CO_2_ conditions. In light of the severely impaired photosynthetic parameters of this mutant, correspondingly massive changes were expectedly observed at the metabolite level (Figure 8, full data set in Table S3). First, leaf glycolate content was four-fold (day) or even nine-fold (night) elevated in 1% CO_2_ in comparison with the wild type and even more so in comparison with *hpr1*. The high day-levels decreased in ambient air and resembled wild-type levels at day five in LC. Glycine levels were also much higher than in the wild type, both during the day and night. They were not very different at HC and LC and remained at a relatively constant high level throughout the experiment, with the exception of an intermittent strong increase on day one. These high levels of glycolate and glycine in leaves of the HC-grown *pglp1* mutant are not to be anticipated from the direction of carbon flow through the photorespiratory cycle and therefore indicate extensive metabolic reprogramming. Glycerate, which is a more downstream intermediate of the photorespiratory pathway, also accumulates at HC but to lower levels than glycine and without the initial intermittent increase observed with glycine on day one in LC. Hydroxypyruvate levels, on the other hand, were relatively low in *pglp1* at HC and LC but increased rather than decreased (as in the wild type) during five days in ambient air.

Among the metabolites of the TCA cycle, both day and night levels of succinate were distinctly elevated at HC relative to the wild type control under otherwise identical conditions and remained high at LC. By contrast to succinate, citrate levels were similar to the wild type but intermittently accumulated to three-fold higher level on days one and three but remained about unchanged during the nights in low CO_2_. The opposite trend prevails for α-ketoglutarate, which decreased below the wild-type level on day one followed by a gradual but moderate increase during the following days. A similar increase occurred over the following nights, but α-ketoglutarate levels of the mutant were always below the corresponding wild-type levels. Quantitatively similar, day-levels of fumarate and malate were also lower than in the wild type, both under high or low CO_2_ and in light or dark conditions, respectively. Taken together, these effects suggest reduced TCA cycle flow at the complex II (succinate dehydrogenase) step, which links the pathway to mitochondrial electron transport. Notably, γ-aminobutyric acid (GABA) was also elevated, up to ten-fold, under all these conditions and over the entire period analysed. Since glutamate is strongly reduced, this could possibly indicate backflow from the elevated succinate content [39].

Concerning key metabolites of nitrogen and amino acid metabolism, in addition to α-ketoglutarate and apart from the less significant changes observed in the levels of glutamate, relative day aspartate contents were reduced already in high CO_2_ and went down to even lower levels on day one of low CO_2_ before they eventually recovered on day five. This is a clear day effect since the aspartate content was relatively stable during the night. While effects on these metabolites were all relatively small, they were much higher for asparagine, arginine, and ornithine. All three compounds rapidly increased in *pglp1* under low-CO_2_ conditions, both during the day and the night but with a much stronger night effect at least for ornithine. A similar trend was observed with isoleucine, lysine, valine, and tyrosine, which also showed a very strong accumulation under low-CO_2_ conditions. With the exception of lysine, the highest accumulation occurred during the night. Alanine showed a less strong accumulation with prolonged growth in low CO_2_; however, this amino acid showed the highest accumulation in high CO_2_. Finally, dehydroascorbate levels were much lower than in the wild type (Table S3).

## Discussion

Traditionally, photorespiration is described as a pathway that is attached to the Calvin-Benson cycle to convert 2PG back to 3PGA but without much interaction to other areas of cellular metabolism except ammonia assimilation. This view has changed in recent years when, for example, links to energy metabolism [40], glutathione biosynthesis [41] and folate interconversion pathways [42] were discovered. Much of the knowledge on photorespiration gained after its initial biochemical dissection [43], [44] was based on the analysis of knockout mutants, which are now available for nearly all known constituent enzymes in Arabidopsis. Most of these mutants were only individually characterized in different laboratories and parallel investigations on several mutants were a rare exception. It is natural that the applied experimental conditions were often not fully comparable during this decades-spanning research.

In this study, we attempted a direct comparison of the wild type of Arabidopsis with four photorespiratory mutants to examine the plasticity of the photorespiratory pathway and possibly identify unknown interactions with other areas of cellular metabolism. These five genotypes were grown, side by side, in carefully controlled ‘non-photorespiratory’ HC conditions (1% CO_2_ in air) to a defined developmental stage [25]. Then, individual plants were analysed with respect to a variety of parameters first in HC conditions and then for several days after transition to ambient-air CO_2_ levels.

From a birds-eye perspective, the wild type and *hpr1* did not reveal any clear visible phenotypic alterations after HC-to-LC transition, whereas leaves of the other three mutants became increasingly necrotic (Figure 1, Figure S1, Figure S2) as it was reported before for these and many other photorespiratory mutants, for example [11], [16]. As mentioned above, HPR1 can be efficiently substituted by a cytosolic bypass [21], [24], [45], which well explains the only very moderate low-CO_2_ effects in the *hpr1* mutant. The phenotype of the PGLP-deficient mutant, which is located at the other end of the scale of photorespiratory phenotypes, is clearly different from wild type already at HC. This observation is in some contrast to earlier reports that the phenotypes of another Arabidopsis mutant, *pcoA-1* [18] and a corresponding barley mutant, RPr 84/90 [19] are indistinguishable from the wild type at 0.8–1% CO_2_. However, it is fully supported by PSII photochemical efficiencies. The HC data in Figure 2, which were collected from individual source leaves, clearly confirm impairment of photosynthetic electron transport in *pglp1*. For methodical reasons, we did not determine levels of 2PG, but it appears that the cellular level of this compound is a critical factor and an only slight increase above wild-type concentration impairs normal metabolism. This interpretation is in-line with observations on a glycolate dehydrogenase null-mutant of the cyanobacterium *Synechocystis* PCC 6803 [10]. Despite the operation of a carbon-concentrating mechanism, which strongly inhibits the oxygenase reaction of Rubisco and hence 2PG production, this mutant grows distinctly slower than wild-type cells even in 5% CO_2_ (5%) conditions. Interestingly, in addition to the very strong effects seen in *pglp1*, slight but distinct impairments at HC were also visible in the younger rosette leaves of the other three mutants examined in this study, including *hpr1* (Figure S3). This observation indicates a tissue-dependent pattern of metabolic disturbances in leaves of photorespiratory mutants. Additionally, the data consistently suggest that photorespiration cannot be fully inhibited even by 1% CO_2_, which causes an approximately thirty-fold higher carboxylation/oxygenation ratio of Rubisco relative to air conditions.

Expectedly [46], leaf growth activity of the wild type and somewhat more of *hpr1* was only slightly decreased in LC relative to HC (Figure 5). This indicates that our studies are not biased by a reduction of maximum stomatal conductance as it was observed after long-term adaptation of a number of species to HC [47], [48], but this phenomenon is not yet very well understood and most of the relevant studies concern LC-to-HC transitions on a more moderate scale. Growth of the other three mutants, in particular *pglp1*, responds distinctly stronger to the HC-to-LC transition. Their diel leaf growth patterns were more variable in comparison to wild-type plants at HC. The minor alterations visible in Figure 5 to a higher or lesser degree for the individual mutants may indicate disturbed temporal synchronization of metabolic or source-to-sink transport processes on a diel timescale similar as it was shown with circadian-clock mutants [49]. Taken together, while the diel growth patterns of the mutants did not show very clear alterations, the impairment of mean diurnal and nocturnal leaf growth in LC is evident and results in smaller rosettes some days after transition from HC to LC (Figure 1B). This effect is strongest in *pglp1*, where leaf growth activity ceases immediately after transition from HC to LC (Figure 1B) and does not recover, neither at day or night. The most likely explanation for this extreme feature is the massive negative effect on starch metabolism in *pglp1* (Table 1). Moderate impairments of starch metabolism in photorespiratory mutants have been previously reported [50], [51] and were observed in all mutants examined during this study. This includes *hpr1*, indicating that a fully operational photorespiratory carbon recycling is required for chloroplasts to maintain normal levels of starch synthesis. The effects on starch metabolism in *pglp1*, however, were unexpectedly strong. In a sense, they remind to observations with the starch-less phosphoglucomutase mutant, in which low nocturnal sugar levels rather than high levels in the light trigger responses of diurnal gene regulation [52], [53].

In principle, it is known for decades that the perturbation of photorespiratory carbon flow dramatically restricts photosynthesis. It is an unresolved question though whether this inhibition of photosynthesis is simply caused by drainage of carbon from the Calvin-Bassham cycle [54] or due to the downregulation of specific enzymes, for example by higher levels of intermediates of the photorespiratory pathway. Since starch metabolism was less impaired in *glyk1* and also impaired in *hpr1*, it is not very likely that the strong inhibition in *pglp1* is simply due to drainage of Calvin-Benson cycle metabolites. Instead, the intra-chloroplastidial level of 2PG could be one of the factors controlling photosynthetic carbon-metabolism as a whole, and PGLP activity could be of key regulatory importance in the coordination of photorespiration and photosynthesis. Mechanistically, such control could be exerted via the reported 2PG inhibition of triose-phosphate isomerase [55], [56] and phosphofructokinase [57], which are both central enzymes of photosynthetic carbohydrate metabolism. Alternatively, the very low starch contents of *pglp1* leaves could also indicate elevated diurnal breakdown of transitory starch, but such an explanation would be unsound in light of the very low maltose levels during day and night (Table S3). Hence, our data suggest that the virtually starch-free phenotype of *pglp1* is mainly due to inhibited starch synthesis rather than accelerated degradation.

A very interesting long-term adaptation effect was observed with *shm1* (Figure 3). Following transfer to LC, only the fully developed leaves (source leaves) but not the young leaves (sink leaves) of this mutant displayed a strong visible response. Following initial growth for some weeks in HC, *shm1* was not only able to survive in ambient air but even flowered and produced viable seeds. This is both unexpected and remarkable since *shm1* represents one of the classical photorespiratory mutants [51]. Moreover, overexpression of the second mitochondrial SHM, SHM2, cannot complement the *shm1* allele [23]. We found that this feature very likely results from specific features of the SHM2's mitochondrial targeting peptide, which seemingly does not allow import into mesophyll mitochondria of Arabidopsis leaves, restricting SHM2 to the vasculature [58]. This phenomenon is not yet fully understood but, since there is no other mitochondrial SHM in the *shm1* mutant, the import restrictions for SHM2 could be relaxed after acclimation of *shm1* to HC conditions.

On a smaller time-scale, slow acclimation to low CO_2_ was also visible in photosynthetic gas exchange, in which net CO_2_ uptake increased and CO_2_ compensation points decreased over five days after transition of the wild type to LC (Figure 4). Even higher alterations though in the opposite direction were observed with *glyk1* and *shm1*, whereas the differences in these two parameters between HC and LC conditions were much smaller with *hpr1* and *pglp1*. Particularly the small changes in *hpr1* suggest, but do not fully exclude, that a superimposition by stomata effects is relatively small and that the observed changes are due to metabolic adaptation.

After transition to LC - unsurprisingly - the strongest metabolic changes were observed within the group of photorespiratory metabolites (Figure 6, Figure 7, Figure 8). It is worth noting that the relatively slow speed with which metabolic adjustment to LC proceeds in all lines approximately corresponds to the slow acclimation of photosynthetic gas exchange. In particular, we consider three of the observed effects as most stimulating.

First, the PGLP-deficient mutant accumulates glycolate to four-fold (day) and nearly ten-fold (night) higher levels at 1% CO_2_. These data confirm the above conclusion that photorespiration is not fully inhibited at 1% CO_2_. In quantitative terms, it shall be stressed that these and all other metabolite data represent relative changes and do not allow any conclusion on absolute levels.

Second, day glycolate levels consistently showed a clear decrease on day one in LC with a small but steady increase during the following days, with the exception of further decreasing values in pglp1. The initial drop was most pronounced in the wild type and *hpr1* but significant also in *pglp1*. This is an intriguing observation since it cannot be easily explained with the assumption that photorespiration is an essentially unregulated pathway. It is reasonable to assume that transfer from HC to ambient air will immediately trigger 2PG production and subsequently glycolate synthesis. One possible explanation for the observed decline in glycolate levels could be that glycolate oxidation becomes down-regulated in HC (and possibly in the dark) and light-dependently activated in ambient air [59]. This hypothesis would correspond to the observed massive rise of dehydroascorbate in the wild type on day one at LC and the high levels of dehydroascorbate in *hpr1*. Since this compound is generated, amongst other processes, during capture of reactive oxygen species (ROS) in the ascorbate-glutathione cycle [60], [61], the observed changes indicate a massive alteration in the cellular redox balance during HC-to-LC transition including the induction of ROS. Such induction could occur due to several reasons including an intermittent massive increase in H_2_O_2_ production by glycolate oxidase. Interestingly, it was recently shown that that all five members of the Arabidopsis glycolate oxidase family are important for non-host disease resistance [9], which includes regulation of biosynthesis and probably activity.

Third, *hpr1* shows very high leaf glycine levels with a massive about 80-fold intermittent rise on day one after HC-to-LC transition. Over the following days, this peak drops sharply to then increase again. A similar but distinctly less pronounced dynamics of glycine levels is visible in *pglp1*. Increases in daily leaf glycine levels following transition from high to low CO_2_, as shown in Figure 6 for the Arabidopsis wild type, are to be expected and were often observed before. Barley leaves, for example, accumulate about 40% more glycine in air than in 0.7% CO_2_ [12], and the steady-state glycine content of potato and wheat leaves varies strongly depending on environmental conditions [62]. By contrast to these predictable changes, the remarkable massive intermittent rise immediately after HC-to-LC transition of *hpr1* though has not been observed before and cannot be explained with present knowledge of the biochemistry of photorespiratory metabolism. Since this peak was not observed with the wild type, it cannot simply be related to a ‘photorespiratory shock’ but is related to the function(s) of HPR1. We speculate that at least one of the two glycine-to-serine conversion reactions could be down-regulated at very low photorespiratory flux (1% CO_2_). Up-regulation could then require high photorespiratory flux (at LC) in combination with fully functional peroxisomal recycling of NADH to NAD^+^ (by HPR1). Since the peroxisomal membrane forms a permeability barrier for NAD(P)^+^ and NAD(P)H, it is thought that HPR1 and malate dehydrogenase are important enzymes for the redox equilibration within the peroxisomes [38], [63], [64]. HPR1 hence has an extraordinary position relative to other enzymes of the photorespiratory core cycle not only because it can be efficiently circumvented by the cytosolic enzyme HPR2 but also in light of its role for peroxisomal and possibly cellular redox homeostasis. Therefore, it is tempting to speculate that glycine decarboxylase (GDC), beyond the established substrate-level regulation via NAD^+^ [65], [66], could be under direct redox-control. This would correspond to the opposite trends observed with dehydroascorbate in the wild type and *hpr1* discussed above, which also indicate that the deletion of HPR1 disturbs cellular redox homeostasis.

The coordination of photorespiration with the TCA cycle has been presumed for some time, but our understanding of how this is precisely achieved is currently fragmentary [37]. Since the activity of several enzymes of this pathway is affected by metabolites produced or consumed during photorespiration, for example [67], it is not too surprising that there were also moderate effects of the HC-to-LC transition on the TCA cycle in the wild type and in *hpr1*. On the other hand, a massive reprogramming takes place in *pglp1* at HC and even more so at LC: the TCA cycle becomes disrupted and a massive accumulation of branched-chain and aromatic amino acids occurs. All these changes indicate up-regulation of alternative pathways of respiration relying on protein, fatty acid and maybe also chlorophyll degradation [68]. It is also remarkable that several amino acids accumulate to very high levels in *pglp1*. These values indicate an additional block in the degradation of branched-chain and aromatic amino acids and therefore a possible link of photorespiration to this part of metabolism. Given the close relationship between photorespiration and respiration, these pathways could be invoked to maintain electron supply to the mitochondria. In addition, fumarate and malate are strongly decreased and a function as alternative respiratory metabolites under carbon starvation has been described [68], [69], supporting this notion. Moreover, succinate and GABA are consequently elevated in *pglp1*, indicating an activation of the GABA shunt to replenish the TCA cycle [70]. These data from metabolic profiling of the transition from high to low CO_2_ further support our assumption of a key position of PGLP1 in the concerted regulation of photosynthetic-photorespiratory metabolism and possibly beyond. Currently, the observed high levels of glycolate and glycine in *pglp1* are very difficult to explain; this even more so in light of the low levels of serine, hydroxypyruvate and glycerate at high-CO_2_, which confirm an expectedly low photorespiratory flux under this condition. Possibly, these low levels could be related to alternative routes of serine biosynthesis [71], [72], which would at least explain the nearly unaltered serine levels.

Particularly in light of the observed very massive metabolic impact of the PGLP1 knockout, the interesting question arises whether the photorespiratory pathway or some of its parts are essential for other metabolic processes in addition to its importance for the Calvin-Benson cycle. Our data do not provoke a substantiated new answer to this old question but demonstrate that an undisturbed photorespiratory carbon flow is essential for the ‘normal’ operation of several other metabolic pathways.

### Conclusions

With this side-by-side comparison of mutants impaired in selected individual steps of the photorespiratory pathway, we arrived at five major conclusions:

Firstly, there is not a single photorespiratory phenotype, but rather there are many: the phenotypes of photorespiratory mutants heavily depend on the interrupted reaction and the availability of short-term (*hpr1*) and long-term (*shm1*) compensation processes. In fact, *glyk1* was the only mutant in our set of four which displayed the whole set of ‘classical’ features of a photorespiratory mutant. Additionally, the perturbation of photorespiratory metabolism shows a tissue-dependent pattern.

Secondly, acclimation of the wild type and photorespiratory mutants from high to low CO_2_ typically is not a fast process but requires several days. In case of *shm1*, it can be influenced by the duration of a preceding preconditioning period in high CO_2_.

Thirdly, photorespiratory metabolism is not as isolated as often thought but integrated into whole-plant metabolism in a manifold and complex manner. Metabolic and regulatory links appear to go far beyond the obvious direct interactions with the Calvin-Benson cycle and N-metabolism but include at the very least starch metabolism, the TCA cycle, the metabolism of branched-chain amino acids, and redox metabolism. Therefore, an undisturbed photorespiratory carbon flow is essential for the ‘normal’ operation of these metabolic pathways.

Fourthly, our data support the role of 2PG as a likely key metabolic regulator. This compound is seemingly produced even at 1% CO_2_, indicating that photorespiration is not fully inhibited at this condition.

Fifthly, although photorespiration is driven by light, some important and dynamically varying phenotypic properties such as leaf growth or metabolite contents were shown to be most prominently altered at night. This fact alone demonstrates that photorespiration is a temporally highly organized process which is involved in many regulatory and maintenance processes on a short timescale in a non-intuitive manner. The fine-tuning of this dynamic control however remains to be elucidated at higher resolution in future studies.

## Materials and Methods

### Plant Material and Growth

*Arabidopsis thaliana* ecotype Columbia (Col-0) was used as wild-type reference. The isolation of the individual homozygous mutant lines from SALK lines SALK_ 130837 (*pglp1-1*, *At5g36700*), SALK_ 083735 (*shm1-2*, *At4g37930*), SALK_067724 (*hpr1-1*, *At1g68010*) and SALK_ 085479 (*glyk1-1*, *At1g80380*) has been described before [17], [20], [21], [58]. Seeds were incubated at 4°C for at least 2 days to break dormancy prior to germination. Plants were grown in ambient air (LC, 0.038% CO_2_) or elevated CO_2_ conditions (HC, 1% CO_2_) in controlled environment Percival chambers (10/14 h or 16/8 h day/night-cycle, 20/18°C, ∼120 µmol·m^−2^·s^−1^ irradiance) on a 4∶1 mixture of soil (Type Mini Tray; Einheitserdewerk, Uetersen) and vermiculite and regularly watered with 0.2% Wuxal liquid fertilizer (Aglukon). If other conditions were not specifically mentioned, we analysed leaves from plants at growth stadium 5.1 according to Boyes et al. [25]. For the HC-to-LC transitions, CO_2_ levels were reduced in a controlled manner without transfer to different growth cabinets.

### PSII Photochemical Efficiency and Gas Exchange

For fluorescence-imaging and the determination of F_v_/F_m_ ratios, we used an Imaging PAM (M-series, Walz) according to Schreiber et al. [73]. Measurements were performed before (HC control) and one, three and five days after HC-to-LC transition (LC) as described in Fahnenstich et al. [74]. Net-photosynthetic rates (A, at 380 µL L^−1^ CO_2_) and CO_2_ compensation points (Γ_21_) were measured at 21% O_2_ with a Licor-6400 (LICOR, Lincoln, NE, USA) using fully developed rosette leaves before and after transition to ambient air. Photosynthetic photon flux density was 200 µmol m^−2^ s^−1^ (red/blue LED light source), and the leaf temperature was 25°C. Γ_21_ values were calculated from A/c_i_ curves by regression analysis in the linear range of the response curve (400, 300, 200, 100, 50, and 400 µL L^−1^ external CO_2_). Plants were adapted to the respective conditions for 10–15 minutes before measurement.

### General and Diel Growth Analysis

General growth of plants grown in HC and LC conditions was analysed non-invasively by monitoring the projected area of complete rosettes of wild-type and mutant populations using the automated high-throughput phenotyping platform GROWSCREEN FLUORO as described previously [27]. Diel leaf growth patterns of plants from the same populations as used for general phenotyping and starch analysis were monitored with high spatial and temporal resolution using the DISP method as described elsewhere in more detail [31]. After one day of acclimation in HC conditions, diel leaf growth was recorded for leaf number 10 of every plant with leaves less than 50% fully grown with a length of 6–12 mm (typical sink leaves). Image acquisition was performed using a set of progressive CCD cameras (Sony XC55 or XC75, Sony, Cologne, Germany) equipped with manually focused standard lenses and an infrared interference filter. Constant day and night illumination was provided using six clusters of infrared diodes (940 nm, Conrad Electronics, Hirschau, Germany). Gray-value images were taken every 90 s and image sequences used to calculate diel growth patterns by calculation velocities from moving visible structures as described before [31]. Relative growth rates (RGR) in % h^−1^ were calculated as divergence from estimated velocity fields by selecting an area of interest (AOI) on the leaf surface within the first image of the acquired image sequence. Structures within this AOI were tracked over 48 h before and 48 h after HC-to-LC transition along the complete image sequence to calculate RGRs in high temporal resolution. The data points in Figure 5 the mean RGR ± SE of the one-hour average RGR values of the preceding hour measured with one leaf each from five individual plants. Grey (night) and white (day) mark the photoperiodic phases.

### Starch Analysis

After two days in LC, leaf samples were harvested at the end of the night (EON), the middle (MOD) and the end of the day (EOD). Samples were weighed, immediately frozen in liquid nitrogen and stored at −80°C until analysis. They were then repeatedly extracted in 400 µl 80% ethanol (2 mM HEPES) at 78°C and the residues subjected to quantitative starch analysis (in hexose units) as described in Walter et al. [46], using a coupled enzymatic assay [75] in a micro-plate absorption spectrophotometer (HT II Plate reader; Anthos Mikrosysteme GmbH, Krefeld, Germany).

### Metabolomics

Metabolite analysis was performed using fully expanded rosette leaves from plants at developmental stage 5.1 [25]. A first set of samples was taken under 1% CO_2_ (HC control) and more sets after transition to ambient air at days 1, 3 and 5 after shifting (LC), 2 h before and 4 h after light was switched off. Samples were immediately frozen in liquid nitrogen and stored at −80°C until analysis. Fifty mg of each sample were extracted and analysed as described previously [76].

### Accession Numbers

Sequence data related to this article can be found in the EMBL/GenBank data libraries under accession number(s) *At5g36700* (PGLP1), *At4g37930* (SHM1), *At1g68010* (HPR1), and *At1g80380* (GLYK).

## Supporting Information

Figure S1 **Phenotypes before and after transition from high CO_2_ to ambient air (short days, 10/14 h).** Plants were grown in high CO_2_ (1%) with a 10/14 h day/night cycle. After reaching developmental stadium 5.1 (about 8 weeks), CO_2_ concentration was reduced to air levels and plants monitored. Individual panels show two representative plants from each line (*hpr1*, *glyk1*, *shm1*, or *pglp1*) next to two wild-type plants (Col-0) grown under identical conditions. Photos were taken before and 3, 5, 7 and 14 days after transition to ambient air.
(TIF)

Figure S2 **Phenotypes before and after transition from high CO_2_ to ambient air (long days, 16/8 h).** Plants were grown exactly as described in the legend to Figure S1 but with a 16/8 h photoperiod and only for 6 weeks since developmental stage 5.1 was reached earlier in long days. The display format also corresponds to Figure S1.
(TIF)

Figure S3 **PSII fluorescence imaging before and after transition to ambient air.** Plants were grown exactly as described in the legend to Figure S1. At the indicated times after transition to ambient CO_2_, plants were dark-adapted for 10 min and fluorescence images collected. Images are normalized to the F_v_/F_m_ color bar at the bottom at the figure. Shown is one representative image from a total of five examined individuals per line and time point.
(TIF)

Table S1 Metabolic response of Arabidopsis wild type after transition to ambient air.
(XLSX)

Table S2 Metabolic response of *hpr1* after transition to ambient air.
(XLSX)

Table S3 Metabolic response of *pglp1* after transition to ambient air.
(XLSX)
