# Supplementary figures and images for: High-to-Low CO2 Acclimation Reveals Plasticity of the Photorespiratory Pathway and Indicates Regulatory Links to Cellular Metabolism of Arabidopsis
Source: PLoS One. 2012 Aug 17;7(8):e42809. doi: 10.1371/journal.pone.0042809 (PMC3422345; doi:10.1371/journal.pone.0042809)

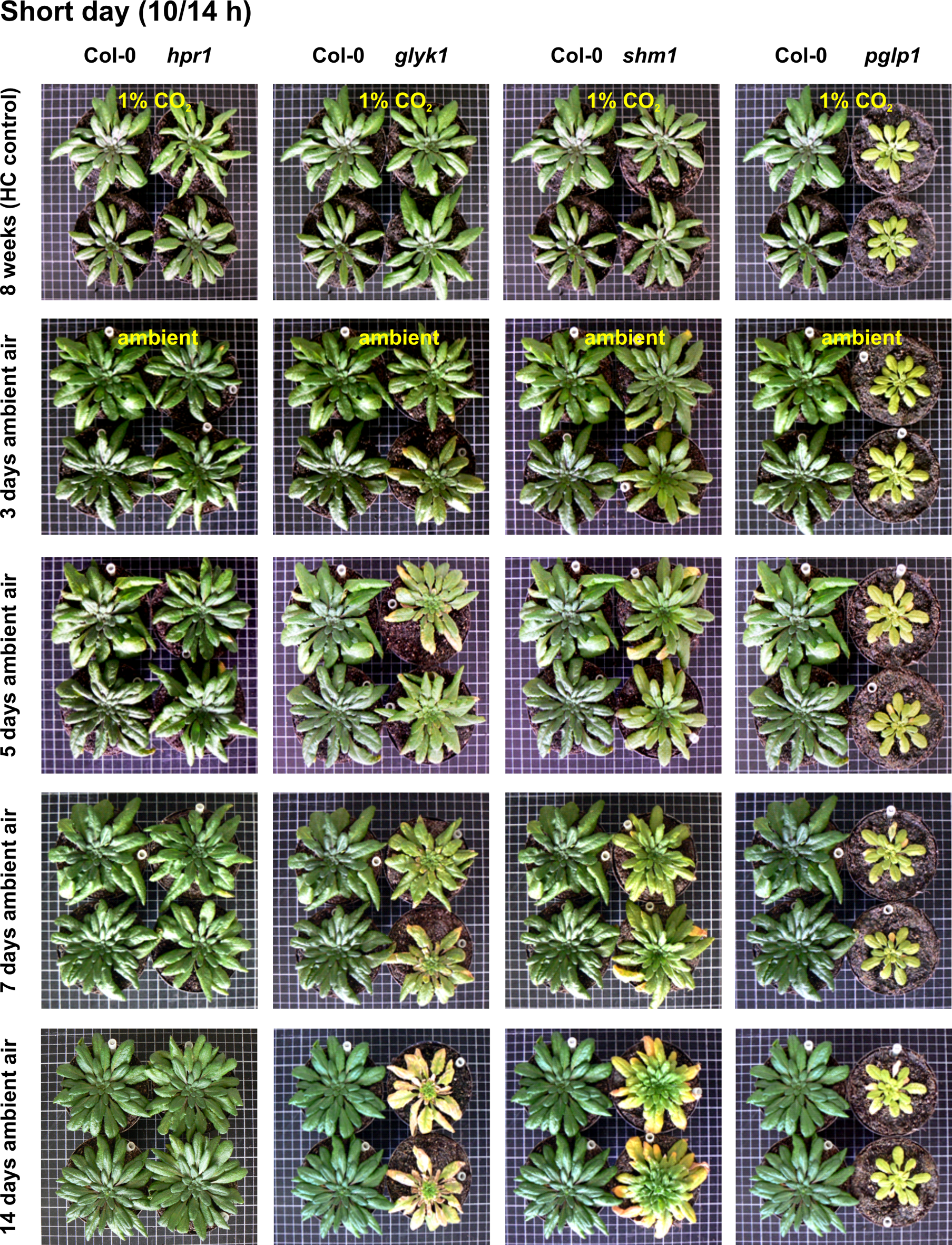

Supplement: Figure S1 — Phenotypes before and after transition from high CO2 to ambient air (short days, 10/14 h). Plants were grown in high CO2 (1%) with a 10/14 h day/night cycle. After reaching developmental stadium 5.1 (about 8 weeks), CO2 concentration was reduced to air levels and plants monitored. Individual panels show two representative plants from each line (hpr1, glyk1, shm1, or pglp1) next to two wild-type plants (Col-0) grown under identical conditions. Photos were taken before and 3, 5, 7 and 14 days after transition to ambient air. (TIF) [file pone.0042809.s001.tif]

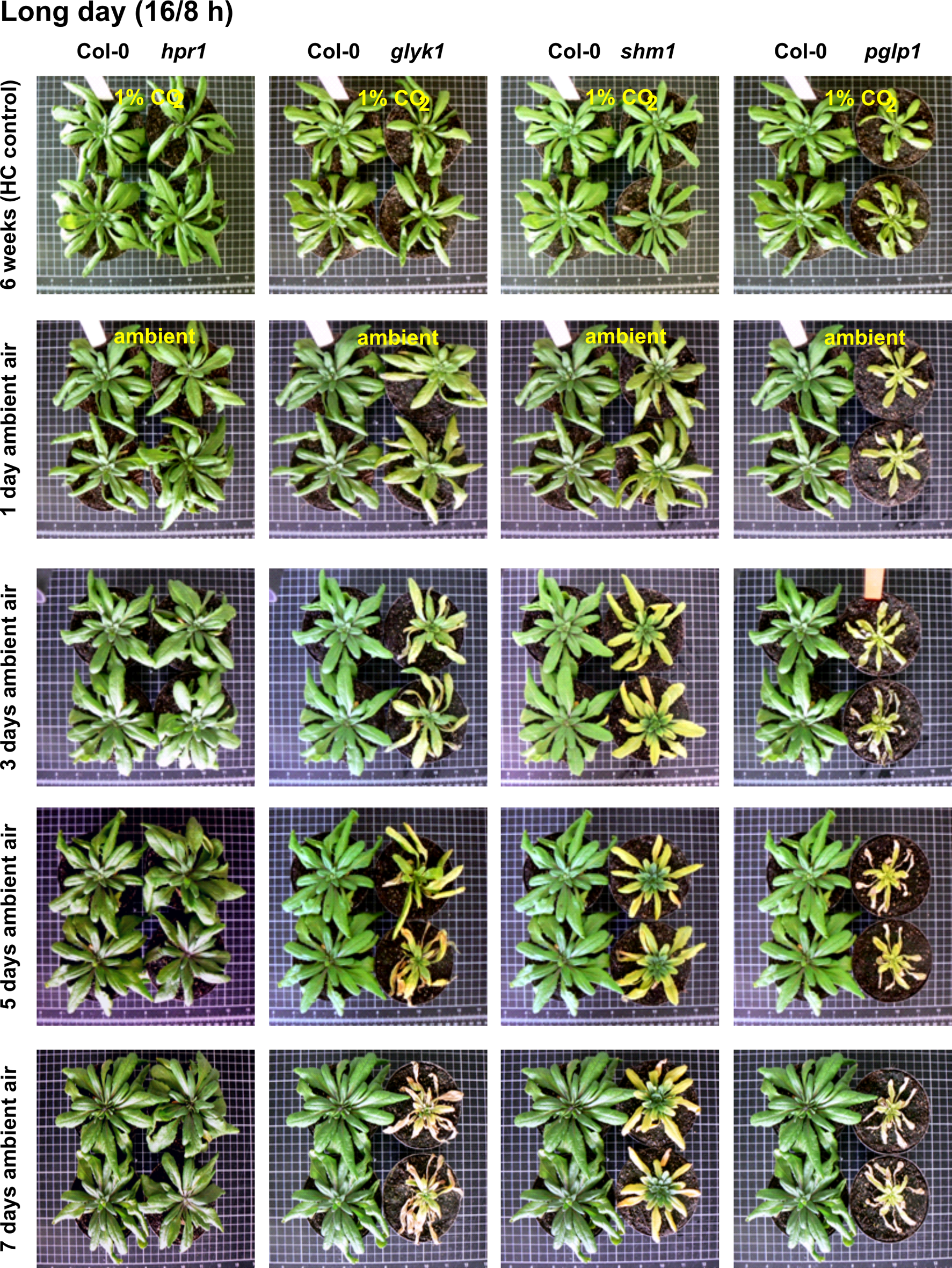

Supplement: Figure S2 — Phenotypes before and after transition from high CO2 to ambient air (long days, 16/8 h). Plants were grown exactly as described in the legend to Figure S1 but with a 16/8 h photoperiod and only for 6 weeks since developmental stage 5.1 was reached earlier in long days. The display format also corresponds to Figure S1. (TIF) [file pone.0042809.s002.tif]

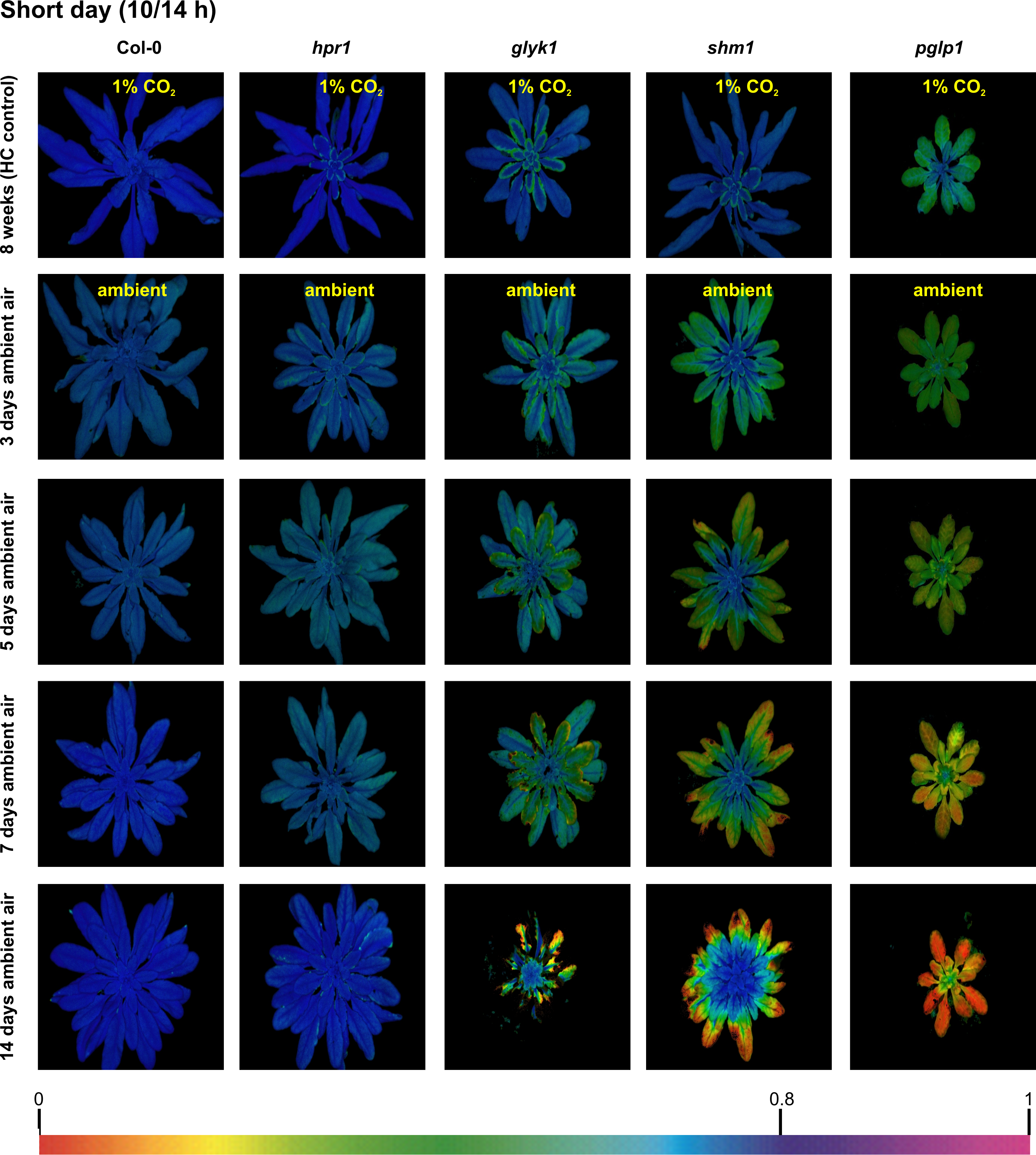

Supplement: Figure S3 — PSII fluorescence imaging before and after transition to ambient air. Plants were grown exactly as described in the legend to Figure S1. At the indicated times after transition to ambient CO2, plants were dark-adapted for 10 min and fluorescence images collected. Images are normalized to the Fv/Fm color bar at the bottom at the figure. Shown is one representative image from a total of five examined individuals per line and time point. (TIF) [file pone.0042809.s003.tif]
